# A Hidden Markov Model for Urban-Scale Traffic Estimation Using Floating Car Data

**DOI:** 10.1371/journal.pone.0145348

**Published:** 2015-12-28

**Authors:** Xiaomeng Wang, Ling Peng, Tianhe Chi, Mengzhu Li, Xiaojing Yao, Jing Shao

**Affiliations:** 1 Institute of Remote Sensing and Digital Earth, Chinese Academy of Sciences, Beijing, China; 2 University of Chinese Academy of Sciences, Chinese Academy of Sciences, Beijing, China; 3 College of Economics and Management, Southwest University, Chongqing, China; Beihang University, CHINA

## Abstract

Urban-scale traffic monitoring plays a vital role in reducing traffic congestion. Owing to its low cost and wide coverage, floating car data (FCD) serves as a novel approach to collecting traffic data. However, sparse probe data represents the vast majority of the data available on arterial roads in most urban environments. In order to overcome the problem of data sparseness, this paper proposes a hidden Markov model (HMM)-based traffic estimation model, in which the traffic condition on a road segment is considered as a hidden state that can be estimated according to the conditions of road segments having similar traffic characteristics. An algorithm based on clustering and pattern mining rather than on adjacency relationships is proposed to find clusters with road segments having similar traffic characteristics. A multi-clustering strategy is adopted to achieve a trade-off between clustering accuracy and coverage. Finally, the proposed model is designed and implemented on the basis of a real-time algorithm. Results of experiments based on real FCD confirm the applicability, accuracy, and efficiency of the model. In addition, the results indicate that the model is practicable for traffic estimation on urban arterials and works well even when more than 70% of the probe data are missing.

## Introduction

Traffic congestion has become a severe problem in metropolises, resulting in widespread wastage of time and energy [1]. Traffic monitoring and estimation is an important method for obtaining information on traffic conditions; thus, it plays a vital role in reducing traffic congestion [2]. Static sensors (inductive loop detectors [3], video cameras [4], etc.), deployed at fixed locations on roads, are used to detect traffic state (e.g., flow velocity and traffic density). However, it is difficult for these traditional approaches to cover all roads because they involve extensive infrastructure deployment and high maintenance costs [5].

With the rapid development of mobile technologies, recent years have witnessed the emergence of a new method known as floating car data (FCD) for collecting valuable real-time information on traffic conditions. In this method, vehicles (e.g. taxis and buses) equipped with global positioning systems (GPS), accelerometers, and other sensors can provide data such as position, velocity, and acceleration of the vehicle. FCD is not as expensive as traditional data acquisition methods because it requires no dedicated infrastructure. Moreover, it has the potential to provide good spatiotemporal coverage of the transportation network and useful data given a certain penetration rate in the population [6, 7].

In some previous studies, FCD has been used to estimate traffic conditions on highways, providing good results with a low penetration rate (1%–3%) [7–9]. Compared to traffic estimation on freeways, traffic estimation on arterials is more complex because of the traffic lights and intersections, and it requires a greater number of samples for analysis. Several researches have discussed the minimum penetration rate required. For example, Breitenberger et al. [10] proposed a penetration rate of 10% on arterial and urban roads. In addition, Vandenberghe et al. [11] discussed the maximum sample interval and the maximum transmission interval of aggregated samples, where the former defines the time between two consecutive FCD samples captured by the same floating car and the latter defines the time between two consecutive server uploads of all new samples by a floating car.

However, it is difficult for FCD to meet the sampling requirements in practice, and the distribution of observed probe data may be sparse and uneven. Traffic state estimation using sparse probe data has not been explored extensively. Herring et al. [12] proposed a probabilistic modeling framework for estimating arterial travel time distribution using sparse probe data. They modeled the evolution of traffic states as a coupled hidden Markov model (HMM), in which the traffic states of nearby road segments are correlated and evolve over time in a Markov manner. The present study differs from their study in that it considers links with similar traffic conditions instead of adjacent links of the road network, which may improve the modeling accuracy. Yanmin et al. [13] revealed the hidden structures within the traffic conditions of a road network using principal component analysis (PCA) and proposed a compressive sensing-based algorithm for obtaining the missing traffic conditions. However, they simply developed an offline data analytics algorithm that cannot be applied to real-time traffic estimation.

The present study proposes an HMM-based model that focuses on overcoming the problem of data sparseness for traffic estimation using FCD. It is assumed that the traffic state of a road segment is invisible and that each road segment belongs to a cluster of road segments having similar traffic characteristics. The traffic conditions of the other road segments in the cluster are considered as observations, based on which an HMM can be constructed. An algorithm based on clustering and pattern mining is proposed to find all road segment clusters in which segments have similar traffic characteristics, and a multi-clustering strategy is adopted to achieve a trade-off between clustering accuracy and coverage. Through data analysis, two exponential distribution functions are used for computing emission probability and transition probability. Finally, a real-time estimation algorithm is developed for online traffic application. The results of extensive experiments conducted using real floating car data show that our model works well even when more than 70% of the probe data are missing.

The remainder of this paper is organized as follows. Section 2 describes the problem of traffic estimation using sparse probe data. Section 3 discusses the construction of an HMM-based traffic estimation model, outlines the main steps of the proposed approach, and presents an algorithm for real-time traffic estimation. Section 4 describes the implementation of the proposed model and as well as a case study for assessing the accuracy of the model. Finally, Section 5 summarizes our findings and concludes the paper.

## Problem Description

There are numerous floating cars running on the roads. They upload their state information, such as location, speed, and direction, from time to time. The state of a floating car at time *t* is expressed as *s<id*, *l*, *v*, *t>*, where *id*, *l*, and *v* denote the ID, location, and speed, respectively, of the vehicle. A road network *G* is divided into a set of road segments, *R = {r*_*n*_ *|n = 1*, *2*, *…*, *N}*, by intersections. A map-matching algorithm is used to find the road segment on which the vehicle is traveling at time *t*. In order to facilitate statistical analysis, a set of predefined time slots, *T = {t*_*m*_ *|m = 1*, *2*, *…*, *M}*, instead of continuous time, is employed for traffic condition estimation. Then, the state of vehicle *s* is converted into *s<id*, *r*, *v*, *t>*.

In the field of traffic engineering, several metrics have been proposed for quantifying the traffic condition of a link, such as speed [14], density [15], flow [16], and queues at intersections [17]. Furthermore, Many traffic flow models [18–29] have been proposed to study complex traffic conditions. The present study employs the velocity of the traffic flow on a road segment, as in some previous studies [1, 13, 14, 30, 31]. The floating cars are part of the traffic flow; hence, it is reasonable to consider their speed as the speed of the traffic flow. The speed of the traffic flow on segment *n* at time slot *m*, *x* ^*n*,*m*^, is approximated as the average speed of all vehicles moving within the traffic flow on this road segment at time slot *m*. Then, the traffic condition of the road network can be expressed by matrix *X* as follows:

$$X=\left[\begin{array}{ccc} x^{1,1} & \cdots & x^{1,M}\\ \vdots & x^{r,t} & \vdots \\ x^{N,1} & \cdots & x^{N,M} \end{array}\right]$$ (1)

**Fig 1:**
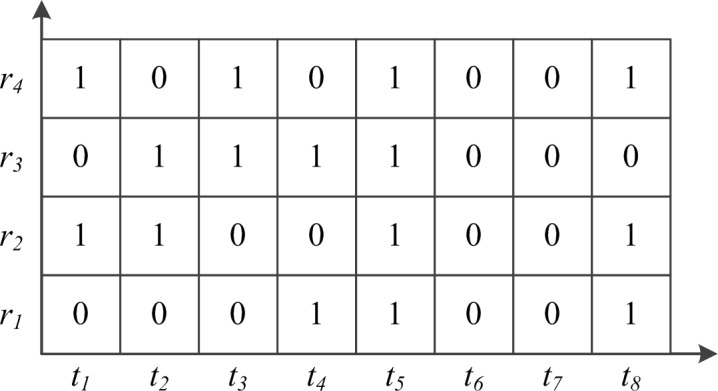
Traffic condition sequences. The value 1 indicates the presence of sample data and 0 indicates the absence of sample data at a time slot.

Here, the row *X*_*r*_ *= {x* ^*r*,*m*^ *|m = 1*, *2*, *…*, *M}* represents the traffic condition sequence of road segment *r* over time. Because of the randomness and unevenness of the floating car data, it is difficult to obtain a complete traffic condition matrix, as there are many spatiotemporal vacancies with no probe measurements. As shown in Fig 1, for the traffic condition sequence of a road segment, there may be some sub-sequences without sample data. Hence, in this study, the main objective of traffic estimation is to estimate the values of these missing states, which can approximate the true states.

## Methods

### Estimation model based on HMM

In this paper, an HMM-based estimation model is proposed to estimate missing traffic state sequences. An HHM, which is based on the concept of Markov process and Markov chain, is characterized by five elements: observation, hidden state, state transition probability, emission probability, and initial state.

The first step in HMM construction is to establish the observations and hidden states of the model. In this study, the traffic condition of the target road segment *r* at time slot *t* is the hidden state *x* ^*r*,*t*^. It is assumed that the road segment *r* belongs to a cluster *C* in which all road segments have similar traffic characteristics. Then, the observations *y* ^*r*,*t*^ are defined as the traffic conditions of the other road segments in the cluster. In some previous studies [16, 32], adjacent road segments have been assumed to be correlated with each other. However, in practice, such an assumption may be not very accurate. A method based on clustering and frequent pattern mining is proposed in Section 3.2 in order to find clusters having road segments with similar traffic characteristics. In this study, speed, which is a continuous variable, is employed as the traffic condition; thus, the hidden state has an infinite number of values. Therefore, it is necessary to select finite candidate states for the HMM process. The state value range at *t* can be limited according to the observation *y* ^*r*,*t*^ and previous state *x* ^*r*,*t-1*^; then, the range should be discretized to a candidate state set *CS* ^*r*,*t*^ *= {x*_*i*_^*r*,*t*^ *|i = 1*, *2*, *…*, *k}*.

The emission probability, *Pr(y* ^*r*,*t*^ *|x* ^*r*,*t*^ *)*, is the likelihood of observing the traffic condition *y* ^*r*,*t*^ conditional on the traffic condition *x* ^*r*,*t*^ being the true condition of the road segment *r* at time slot *t*. The transition probability, *Pr(x* ^*r*,*t*^, *x* ^*r*,*t+1*^ *)*, is the probability that the traffic condition of the road segment *r* will transform from a state *x* ^*r*,*t*^ at time *t* to another state *x* ^*r*,*t+1*^ at time *t+1*. The methods for measuring and calculating the emission probability and transition probability are discussed in Section 3.3.

**Fig 2:**
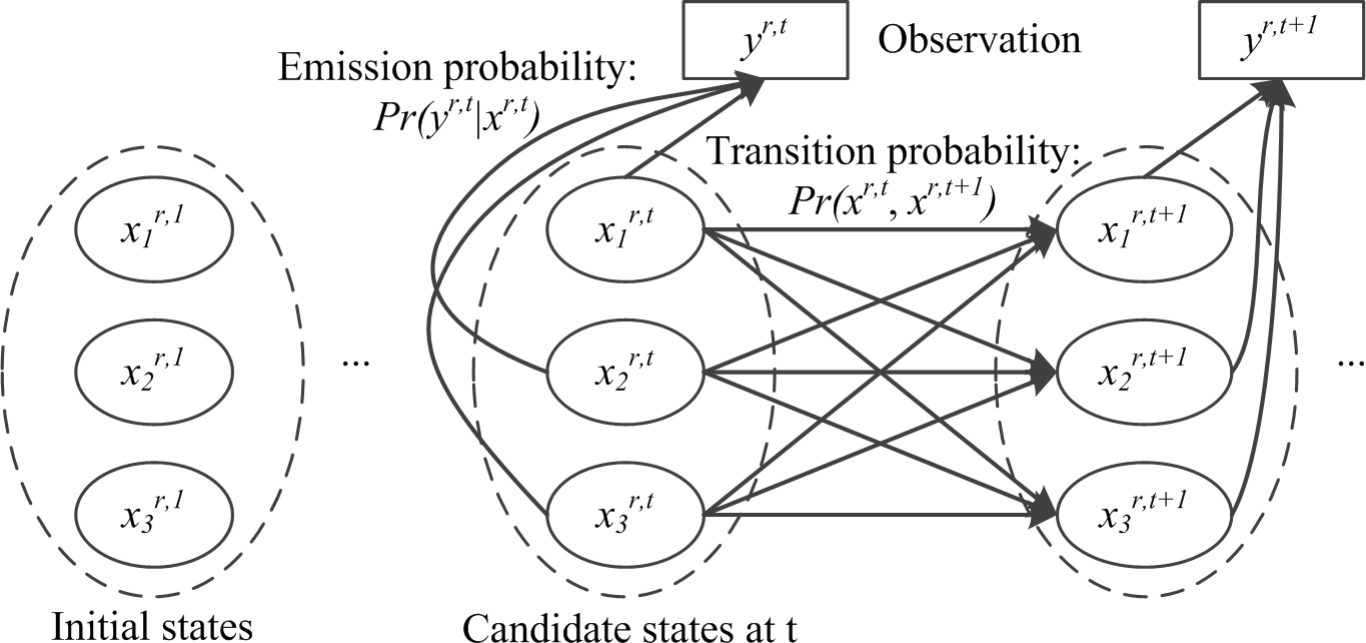
HMM process of traffic condition estimation.

The HMM sequentially generates candidate traffic condition sequences and evaluates them on the basis of their likelihood, which is measured by the joint probability (Fig 2). Past hypotheses of the solution are extended to account for new observations over time. Then, the surviving sequence with the highest joint probability is selected from among the remaining candidates of the previous stage as the final solution. The joint probability is expressed as

$$J^{r,t+1}=Pr(y^{r,t+1}|x^{r,t+1})\;\underset{x^{r,t}\in {\mathrm{CS}}^{r,t}}{\max}\left\{Pr(x^{r,t},x^{r,t+1})J^{r,t}\right\}$$ (2)

where *J* ^*r*,*1*^ *= Pr(y* ^*r*,*1*^ *|x* ^*r*,*1*^ *)* and *CS* ^*r*,*t*^ denotes the set of candidate states of the road segment *r* at time slot *t*. After the HMM process, the last traffic condition sequence with the maximum joint probability can be found: $x^{r,T}={argmax}_{x^{r,T}\in {CS}^{r,T}}\{J_{T}\}$. Then, the system works backwards to find the traffic condition sequence *x*_*T-1*_, *…*, *x*_*1*_ of the road segment.

### Traffic similarity analysis

#### Clustering analysis

In this study, the traffic condition sequence, *X*_*r*_ *= {x* ^*r*,*t*^ *|t = 1*, *2*, *…*, *M}*, is considered as the traffic characteristic of the road segment *r* over a period of time. A spectral clustering algorithm is adopted to divide the road segment set into clusters based on historical data, and the road segments in the same cluster have similar traffic characteristics.

In the spectral clustering algorithm, the set of points in an arbitrary feature space can be represented as a complete weighted undirected graph *G(V*, *E)*. The vertices of the graph *G* are the points in the feature space and the weight *w*_*ij*_ of an edge *(v*_*i*_, *v*_*j*_ *)* in *E* is a measure of the similarity between vertex *v*_*i*_ and *v*_*j*_. In this context, we can formulate the clustering problem as a graph-partitioning problem that requires partitions *V*_*1*_, *V*_*2*_ *…*, *V*_*k*_ of the vertex set *V* according to some measure; then, the vertices in any set *V*_*i*_ have a high degree of similarity, and the vertices in two different sets *V*_*i*_, *V*_*j*_ have a low degree of similarity.

For road segment clustering, the road segments are considered as vertices of the graph *G*. The weight *w*_*ij*_ between the road segments *i* and *j* is the difference between the traffic characteristics of the two road segments; it can be expressed by a Euclidean distance as follows:

$$w_{ij}=\sqrt{\sum_{t=1}^{M}{\left(x^{i,t}-x^{j,t}\right)}^{2}}$$ (3)

where *M* is the number of time slots in the traffic condition sequence and *x* ^*i*,*t*^ is the traffic condition of road segment *i* at time slot *t*. A normalized spectral clustering algorithm (Box 1) is constructed according to previous research [33]:

**Box 1. Spectral clustering algorithm.**

Algorithm 1 SpectralClustering: spectral clustering

**Input**: *G(V, E)*: Traffic condition graph; *k:* Number of clusters;

**Output**: *C = {V_1_,V_2_,…V_k_}*: clusters;

1: Get weighted adjacency matrix *W* of *G(V, E)*;

2: Calculate degree matrix *D* of *W*;

3: $L\leftarrow I-D^{-\frac{1}{2}}∙W∙D^{-\frac{1}{2}}$; //Compute normalized Laplacian

3: Compute the first *k* eigenvectors *_1_,… u_k_* of *L*;

4: Let *U* be the matrix containing the vectors *u_1_, … , u_k_* as columns;

5: Use the *k*-means algorithm to cluster *U*, then get the clusters *C*;

6: return *C*;

In practice, it is difficult to determine a suitable number of clusters for road segment clustering. Therefore, a modified clustering algorithm is proposed, and the average weight *w*_*av*_ of a cluster, instead of the cluster number *k*, is set as a constraint for controlling the clustering process. In order to simplify the computation, *w*_*av*_ is defined as the average weight between the centroid of a cluster and other objects. The centroid is given by

$${vc}_{k}=\frac{1}{\left|V_{k}\right|}\sum_{v_{i}\in V_{k}}v_{i}$$ (4)

where *V*_*k*_ denotes the vertices of the *k*-th cluster, *|V*_*k*_ *|* is the number of vertices in *V*_*k*_, *v*_*i*_ is the *i*-th vertex of *V*_*k*_, and *vc*_*k*_ is the centroid of *V*_*k*_,. Then, the average weight of *V*_*k*_ can be expressed as

$$w_{av}=\frac{1}{\left|V_{k}\right|}\sum_{v_{i}\in V_{k}}\left\|v_{i}-{vc}_{k}\right\|$$ (5)

In the algorithm (Box 2) based on the constraint *ω*, the vertices of *G* are divided into small clusters step by step, and the cluster whose *w*_*av*_ is greater than the threshold value *ω* should be divided into smaller clusters until the constraint is met. Because clusters with only one object are meaningless, it is reasonable to set a minimum number of objects in a cluster (*N*_*min*_). A small value *k* is set as the number of clusters in every clustering step. In this study, both *k* and *N*_*min*_ are set as 2.

**Box 2. Clustering algorithm based on constraint.**

Algorithm 2 ConstraintClustering: Clustering based on constraint

**Input:** *G(V*, *E)*: Traffic condition graph; *ω*: Threshold value; *k*: Number of clusters at every step; *N*_*min*_: Minimum number of objects in a cluster;

***Output*:** *C = {V*_*1*_,*V*_*2*_,*…V*_*K*_ *}*: *clusters;*

1: **if** *|V|>N*_*min*_ and *|V|>k*

2: *C ←* SpectralClustering(*G*, *k*);

3: *C*_*temp*_ *← ∅*;

4: **for** *i ← 1* to *k* do

5: **if** the average weight *w*_*av*_ of *V*_*i*_ is greater than *ω*

6: Get sub-graph *G*_*i*_ from *G* corresponding to *V*_*i*_;

7: *C*_*temp*_ *← C*_*temp*_ ∪ SpectralClustering(*G*_*i*_, *k*);

8: **else**

9: *C*_*temp*_ *← C*_*temp*_ ∪ *{V*_*i*_ *}*;

10: **end if**

11: **end for**

12: *C ← C*_*temp*_;

13: **else**

14: *C ← {V}*;

15: **end if**

16: return *C*;

#### Pattern mining

**Fig 3:**
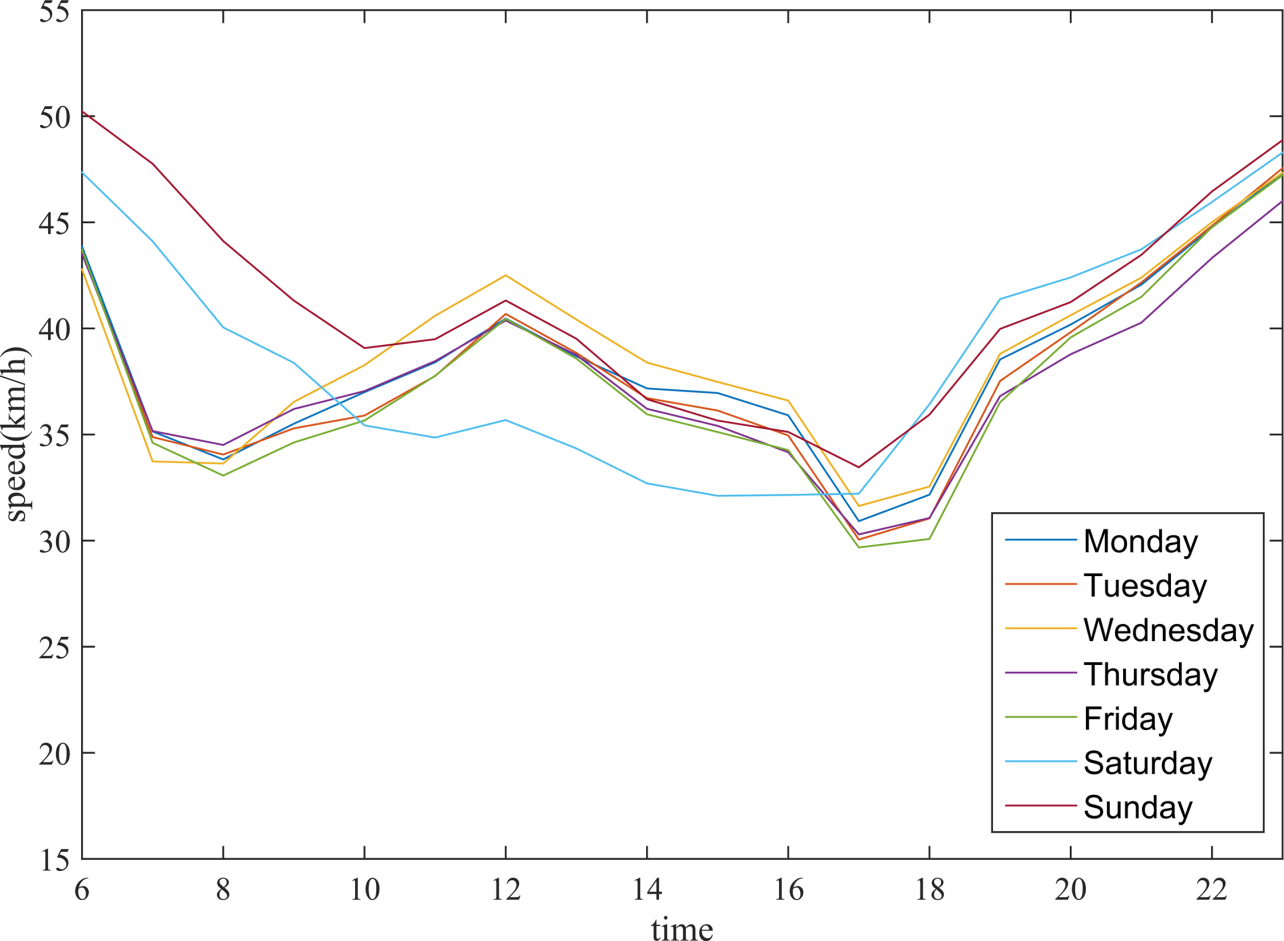
Average speed of arterial roads in Beijing.

To avoid coincidental clusters, it is reasonable to perform clustering multiple times for different days and find frequent clusters with a better representation of traffic similarity between road segments. In general, the traffic condition exhibits different patterns on weekdays and weekends. As shown in Fig 3, the traffic conditions of arterial roads in Beijing have similar characteristics on weekdays but significantly different characteristics on weekends. Therefore, the traffic conditions should be discussed separately.

The road segment set, *R = {r*_*n*_ *|n = 1*, *2*, *…*, *N}*, is divided into cluster set *C*_*d*_ *= {R*_*k*_ *|k = 1*, *2*, *…*, *K}* according to the traffic condition of the *d*-th day using the clustering algorithm proposed in Section 3.2.1. The cluster set list, *L = {C*_*d*_ *|d = 1*, *2*, *…*, *D}*, contains all clusters of the last *D* days (weekdays or weekends) from the target day. The objective of the frequent pattern mining approach adopted in this study is to find the frequent cluster set, *P = {R*_*j*_ *⊂R |j = 1*, *2*, *…*, *J}*, where the cluster *R*_*j*_ appears frequently in *L*. An indicator function is used to indicate whether *R*_*j*_ appears in the cluster set *C*_*d*_:

$$f(R_{j},C_{d})=\left\{ \begin{array}{c} 1\;if\;\exists R_{k}\in C_{d},\;let\;R_{j}\subset R_{k}\\ 0\;if\;∄R_{k}\in C_{d},\;let\;R_{j}\subset R_{k} \end{array}\right.$$ (6)

In this study, the number of times the cluster *R*_*j*_ appears in the cluster set *L* is defined as the support, and it can be calculated as follows:

$$support(R_{j},L)=\sum_{C_{d}\in L}f(R_{j},C_{d})$$ (7)

The frequent cluster must meet the minimum support, *Sup*_*min*_; then, the frequent cluster set can be defined as follows:

$$P(L)=\{R_{j}\subset R|j=1,\;2,\ldots J,and\;support(R_{j},L)\geq {Sup}_{min}\}$$ (8)

**Fig 4:**
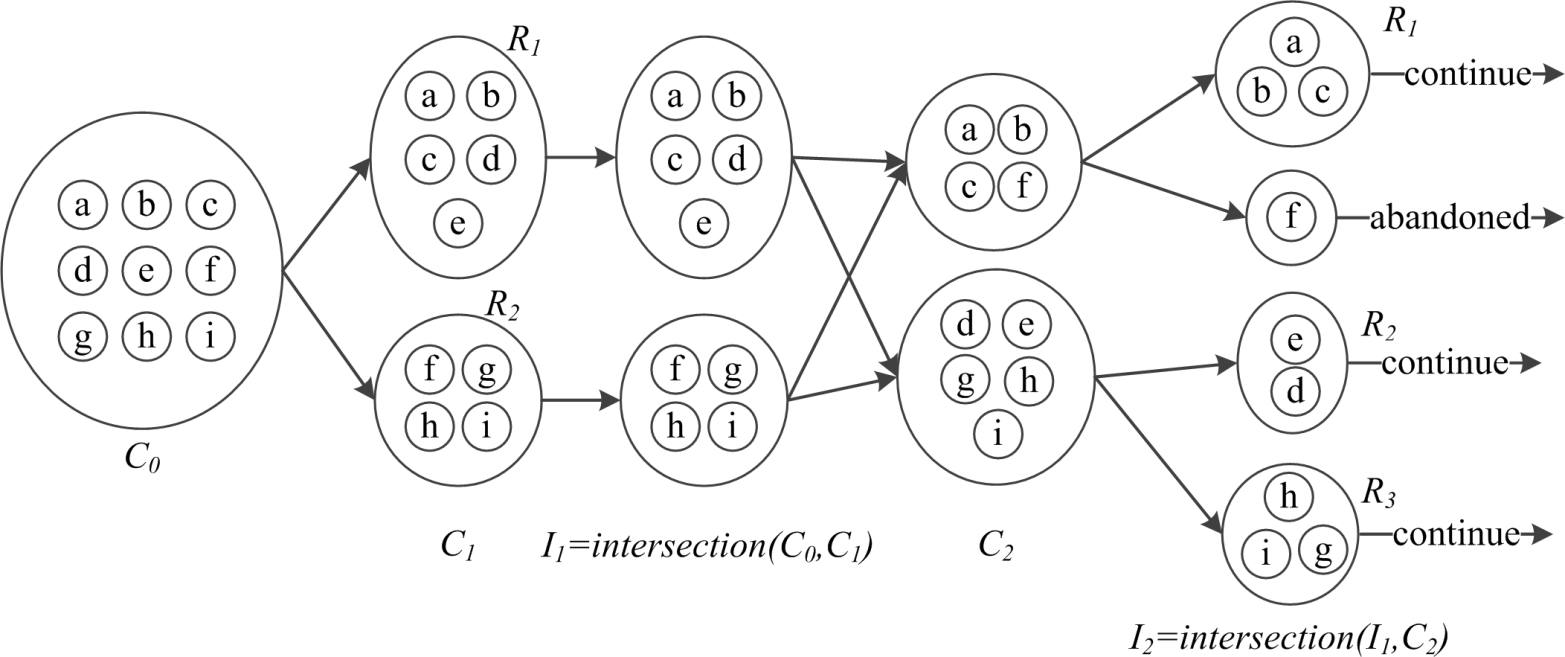
Frequent clusters are obtained by gradual intersection.

It is difficult for traditional pattern mining algorithms (e.g., the Apriori algorithm) to compute and find the frequent clusters, as the number of road segments and clusters is extremely large. To overcome this problem, a frequent pattern mining approach based on intersection is proposed. The intersection between two cluster sets is expressed as

$$intersection(C_{1},C_{2})=\{R_{k}|R_{k}=R_{i}\cap R_{j},R_{i}\subset C_{1},R_{j}\subset C_{2},and\;|R_{k}|>1\}$$ (9)

where *C*_*1*_ and *C*_*2*_ are the cluster sets of two days, *R*_*i*_ and *R*_*j*_ are arbitrary clusters of sets *C*_*1*_ and *C*_*2*_, respectively, and *R*_*k*_ is the intersection of *R*_*i*_ and *R*_*j*_. Note that *R*_*k*_ is meaningful for estimation only if it includes more than one road segment. Therefore, in the intersection process, the cluster *R*_*k*_ will be discarded when *| R*_*k*_ *|<2*. As shown in Fig 4, the frequent cluster set can be obtained by gradual intersection.

For a cluster set list *L*_*i*_, which has *i* cluster sets, the frequent clusters that appear *i* times in *L*_*i*_ can be found by a recursive algorithm based on intersection; this algorithm can be expressed as

$$Pattern(L_{i})=intersection(C_{j},Pattern(L_{i}-C_{j}))$$ (10)

where *C*_*j*_ is an arbitrary cluster set in the list *L*_*i*_; if *L*_*i*_ contains only one cluster set *C*_*j*_, then *Pattern(L*_*i*_ *)* will return *C*_*j*_. In order to find all frequent clusters that appear *i* times in *L*, it is necessary to obtain the combination set, given by

$$L_{com}^{i}=\{L_{i}^{k}|k=1,2,\ldots \;,\left(\overset{\left|L\right|}{\underset{i}{}}\right)\}$$ (11)

where $L_{com}^{i}$ includes all combinations that contain *i* cluster sets of *L*; the number of combinations is (|L|i). Then, the frequent cluster set can be obtained as

$${FC}_{i}={⋃}_{l\in L_{com}^{i}}Pattern(l)$$ (12)

where *FC*_*i*_ contains all frequent clusters whose support is *i*. Then, all frequent clusters that appear more than *Sup*_*min*_ times can be obtained using the following algorithm (Box 3):

**Box 3. Traffic frequent pattern mining algorithm.**

Algorithm 3 TrafficPatternMining: Traffic frequent pattern mining

**Input:** *L = {C*_*1*_, *C*_*2*_, *…*, *C*_*D*_ *}*; *Sup*_*min*_: minimum support

**Output:** *FC = {FC*_*i*_ *|i = Sup*_*min*_, *Sup*_*min*_ *+1*, *…*, *D}*;

1: *FC ← ∅*;

2: **for** *i ←Sup*_*min*_ to *D*

3: *FC*_*i*_ *← ∅*;

4: *L*_*com*_ *← i-combinations from L*;

5: *FC*_*i*_ *←* Get all frequent clusters from (12);

6: *FC ← FC ∪ {FC*_*i*_ *}*;

7: **end for**

8: return *FC*;

#### Multi-clustering strategy

As discussed in Section 3.2.1, the smaller the value of the constraint *ω*, the more similar are the traffic characteristics of the road segments in the same cluster; this may improve the estimation accuracy. However, the frequent cluster set covers fewer road segments because of the more stringent constraint. To resolve this conflict, a multiple-clustering strategy is adopted. Multiple constraints *ω*_*1*_, *ω*_*2*_,*…ω*_*m*_ are selected for clustering and pattern mining; then, a list of frequent cluster sets, *FCL = {FC*_*ω*_ *|ω = ω*_*1*_, *ω*_*2*_, *…*, *ω*_*m*_ *}*, is generated, where *FC*_*ω*_ is the frequent cluster set corresponding to the constraint *ω*. In the process of finding the cluster that contains the road segment *r*, the frequent cluster set with smaller *ω* should be considered first.

### Probability calculation

#### Emission probability

For a road segment *r* that belongs to the frequent cluster *C*, its traffic condition *x* ^*r*,*t*^ at time slot *t* approximates the traffic conditions *y* ^*r*,*t*^ of other road segments in *C*. Thus, the difference *diff* between *x* ^*r*,*t*^ and *y* ^*r*,*t*^ can be adopted to calculate the emission probability *Pr(y* ^*r*,*t*^ *|x* ^*r*,*t*^ *)*. According to observations, the emission probability follows an exponential distribution; hence, it can be calculated as

$$Pr(y^{r,t}|x^{r,t})={\lambda e}^{-\lambda ∙diff}={\lambda e}^{-\lambda \frac{1}{\left|C^{\prime }\right|}\sum_{i\in C^{\prime }}\left|x^{r,t}-x^{i,t}\right|}$$ (13)

where, *λ* is the parameter of the exponential distribution (*λ>0*) and*diff* is the average traffic condition difference between the road segment *r* and road segments in *C' = {r'∈C| r'≠r}*.

In order to find the appropriate frequent cluster *C*, the frequent cluster set *FC*_*ω*_ with a smaller constraint value *ω* should be considered preferentially, and in the frequent cluster set *FC = {FC*_*i*_ *|i = Sup*_*min*_, *Sup*_*min*_ *+1*, *…*, *D}*, the clusters that appear more frequently should be considered first.

#### Transition probability

Through data analysis, in a relatively short time interval, the traffic condition at time slot *t+1* is close to that at time slot *t*. Thus, the traffic state change *∆x = |x* ^*r*,*t*^ *—x* ^*r*,*t+1*^ *|* is employed to measure the state transition. According to observations, the state transition probability follows an exponential distribution, and it can be expressed as

$$Pr(x^{r,t},x^{r,t+1})={\beta e}^{-\beta ∙\Delta x}$$ (14)

where, *β* is the parameter of the exponential distribution (*β>0*).

### Candidate selection

As discussed in Sections 3.2 and 3.3, the state *x* ^*r*,*t+1*^ may approximate the previous state *x* ^*r*,*t*^ and the observations *y* ^*r*,*t+1*^ *= {x* ^*i*,*t+1*^ *|i∈C and i≠r}*, where *C* is the frequent cluster that contains the road segment *r*. Then, the value range of *x* ^*r*,*t+1*^ is set as *[x*_*min*_ *-μ*, *x*_*max*_ *+μ]*, where *x*_*min*_ = *min({x* ^*r*,*t*^ *}*∪*y* ^*r*,*t+1*^ *)*, *x*_*max*_ = *max({x* ^*r*,*t*^ *}*∪*y* ^*r*,*t+1*^ *)*, and *μ* is used to avoid missing valid values. For computational convenience, the range should be discretized to finite candidates denoted by the set

$$CS=\{x_{i}|x_{i}=x_{min}-\mu +i∙\frac{x_{max}-x_{min}+2\mu }{N_{cand}-1},i=0,1,2\ldots ,N_{cand}-1\}$$ (15)

where *N*_*cand*_ is the number of candidates. In order to facilitate algorithm design, the candidate set is *CS* = *{x* ^*r*,*t+1*^ *}* when the state at time slot *t+1* is obtained from the samples. Then, it is not necessary to find the missing state sub-sequences discussed in Section 2; the entire state sequence can be estimated in a single process.

### Real-time algorithm

For the road segment *r* at time slot *t*, a list *PreSList = {Se*_*i*_ *|i = 1*,*2*,*…m}* is used to store previous surviving state sequences. A sequence is denoted by *Se = (SS*, *JP)*, where *SS = {x* ^*r*,*1*^,*x* ^*r*,*2*^,*…*,*x* ^*r*,*t-1*^ *}* stores previous consecutive candidate states and *JP* is the joint probability. Using Algorithm 4 (Box 4), the current candidate sequence list *SList* is obtained according to *PreSList* and the candidate states *CS*_*t*_ of road segment *r* at time slot *t*. Then, the state sequence with the maximum joint probability in *SList* is the optimal solution of road segment *r* at time slot *t*; the sequence is given by *argmax*_*Se∈SList*_ *{Se*.*JP}*. For the first state, the initial joint probability of the state sequence is the emission probability of the candidate states. Obviously, the algorithm can output the estimated states in real time; thus, it is applicable to online application.

**Box 4. Real-time traffic estimation algorithm based on HMM.**

Algorithm 4 TrafficEstimation: Real-time traffic estimation based on HMM

**Input:** *PreSList*: List of surviving sequences of road segment *r* at time slot *t-1*; *t*: time.

**Output:** *SList*: List of surviving sequences of road segment *r* at time slot *t*.

1: *SList ← PreSList*; //*SList* is a current surviving sequence list

2: *CS*_*t*_ *←* Get candidate states; //Discussed in Section 3.4;

3: *SListTemp ← ∅*;

4: **if** *t = = 1*

5: **for** *x* in *CS*_*t*_

6: Construct a new state sequence *Se*; Set *x* as the starting state;

7: *Se*.*JP ← Pr(y* ^*r*,*t*^ *|x)*; //Discussed in Section 3.2;

8: Add *Se* into *SList*;

9: **end for**

10: **else**

11: **for** *x* in *CS*_*t*_

12: *Se* ← *argmax*_*Se*∈*SList*_{*Se*.*JP*∙*Pr*(*x* ^*r*,*t*−1^,*x*)};

13: Set *x* as the *t*-th state of *Se*;

14: *Se*.*JP* ← *Pr*(*y* ^*r*,*t*^|*x*)∙*Se*.*JP*∙*Pr*(*x* ^*r*,*t*−1^,*x*);

15: Add *Se* to the temporary list *SListTemp*;

16: **end for**

17: *SList* ← *SListTemp*;

18: **end if**

19: output *argmax*_*Se*∈*SList*_{*Se*.*JP*}; //Real-time output current solution;

20: return *SList*;

## Results and Discussion

For the experiments, 8559 arterial road segments were selected; the roads cover the main regions of central Beijing. The traffic conditions between 6:00 and 24:00 were considered, and the time was divided into 108 time slots at 10-min intervals (e.g., the first time slot was 6:00–6:10 and the 12^th^ time slot was 7:50–8:00).

The taxi trajectory data in Beijing during November 2012 served as the FCD data, obtained from 12,600 taxis. The data samples of six weekdays were selected for a case study; five of these days were used for frequent cluster mining and parameter estimation, and the remaining day was used to test the estimation model. Before the experiments were performed, the trajectory data were matched to the road network using map-matching methods [34–36], and anomalous samples were eliminated.

The model was implemented using a Java platform on a computer having a quad-core CPU (2.2 GHz) and 8-GB memory.

### Frequent cluster mining

Six constraint values *{*ω |ω = *10*, *15*, *20*, *25*, *30*, *35}* were considered in the clustering analysis stage. The average weight *w*_*av*_ discussed in Section 3.2.1 was employed to measure the degree of similarity, which decreased as *w*_*av*_ increased. As shown in Table 1, the mean *w*_*av*_ of the cluster set and the average number of objects in each cluster, *ON*_*average*_, increase with ω. Clusters having a single object cannot be used for estimation; the proportion of such clusters, *r*_*single*_, decreases as ω increases. For traffic estimation, a perfect cluster set has small average *w*_*av*_, large *ON*_*average*_, and small *r*_*single*_. A cluster set having small average *w*_*av*_ is more likely to have small *ON*_*average*_ and large *r*_*single*_, which confirms the existence of the contradiction discussed in Section 3.2.3. Therefore, it is necessary to adopt a multi-clustering strategy.

**Table 1:** Accuracy and coverage of cluster sets corresponding to different ω.

ω	mean *w_av_*	*ON_average_*	*r_single_*(%)
10	12.29	2.34	21.51
15	12.97	2.69	15.06
20	14.61	3.67	9.21
25	16.58	5.09	4.71
30	18.71	7.29	2.34
35	20.77	11.04	1.03

**Fig 5:**
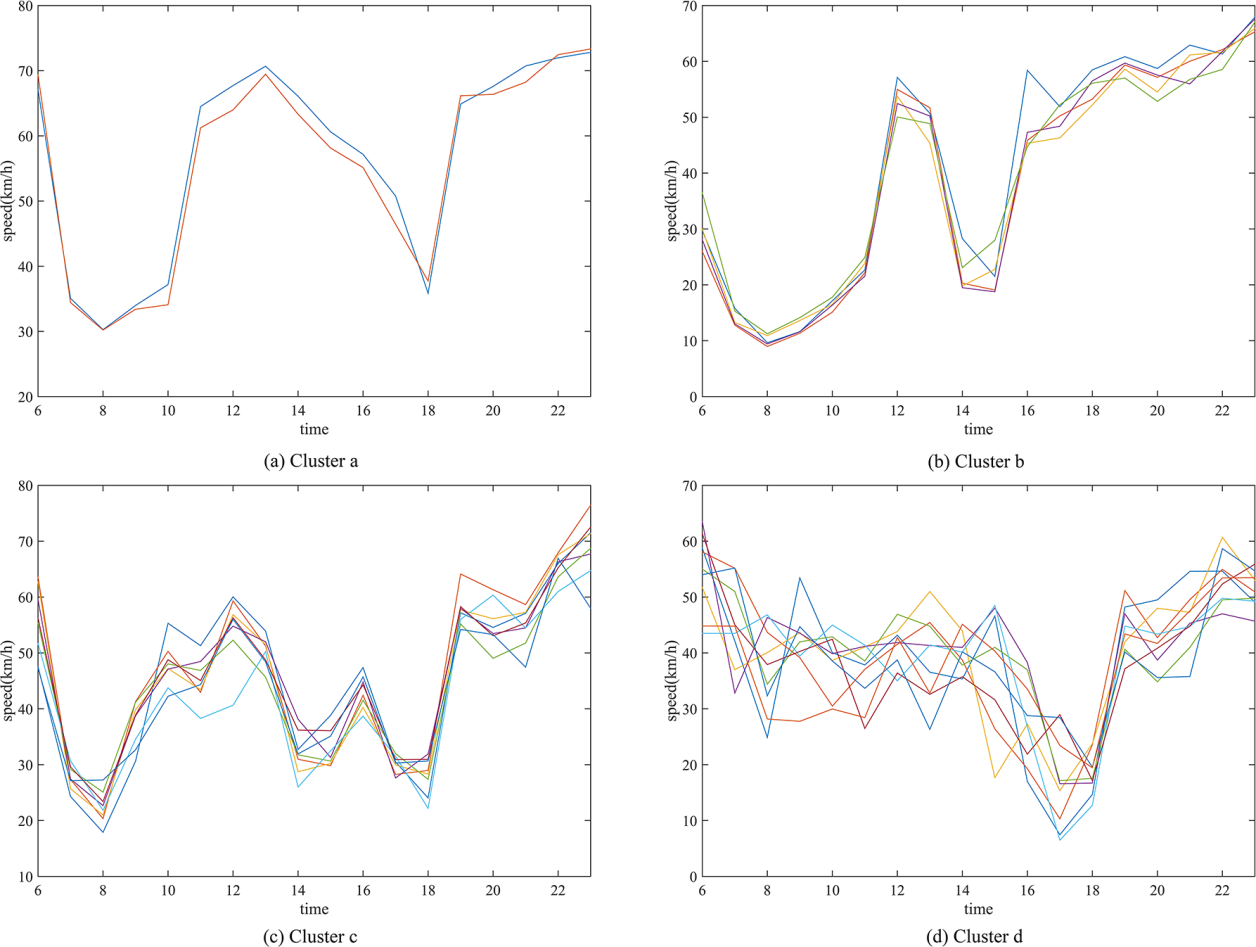
Traffic condition sequences of the road segments in four clusters. (a) The average weight of cluster a is 4.71, (b) the average weight of cluster b is 9.75, (c) the average weight of cluster c is 14.58, and (d) the average weight of cluster d is 24.93.

As shown in Fig 5, the traffic characteristics of the road segments in the same cluster have a very high degree of similarity when the average weight *w*_*av*_ is small, such as clusters a, b, and c. As the average weight *w*_*av*_ increases, the degree of similarity of the cluster decreases and the number of the objects in the cluster increases.

Samples of five days were selected for frequent cluster mining, and the minimum support *Sup*_*min*_ was set as 3. Table 2 lists the coverage rates of the frequent clusters, which is given by the ratio *R*_*cover*_ *= N*_*cover*_ */N*_*total*_, where *N*_*cover*_ is the number of road segments in the frequent cluster set and *N*_*total*_ is the total number of road segments. The coverage rate increases with ω, and the support of the most frequent clusters is less than or equal to 4. When ω increases to 35, the coverage rate of the frequent cluster set reaches 96.76%, which indicates that the set of these ω is sufficient and appropriate for this study.

**Table 2:** Coverage rate of the frequent cluster set for each ω (%).

ω/*support*	≥5	≥4	≥3
10	0.71	4.79	16.75
15	1.33	6.49	20.13
20	1.77	8.8	28.68
25	2.67	14.13	45.76
30	4.84	26.04	75.58
35	7.2	39.1	96.76

**Fig 6:**
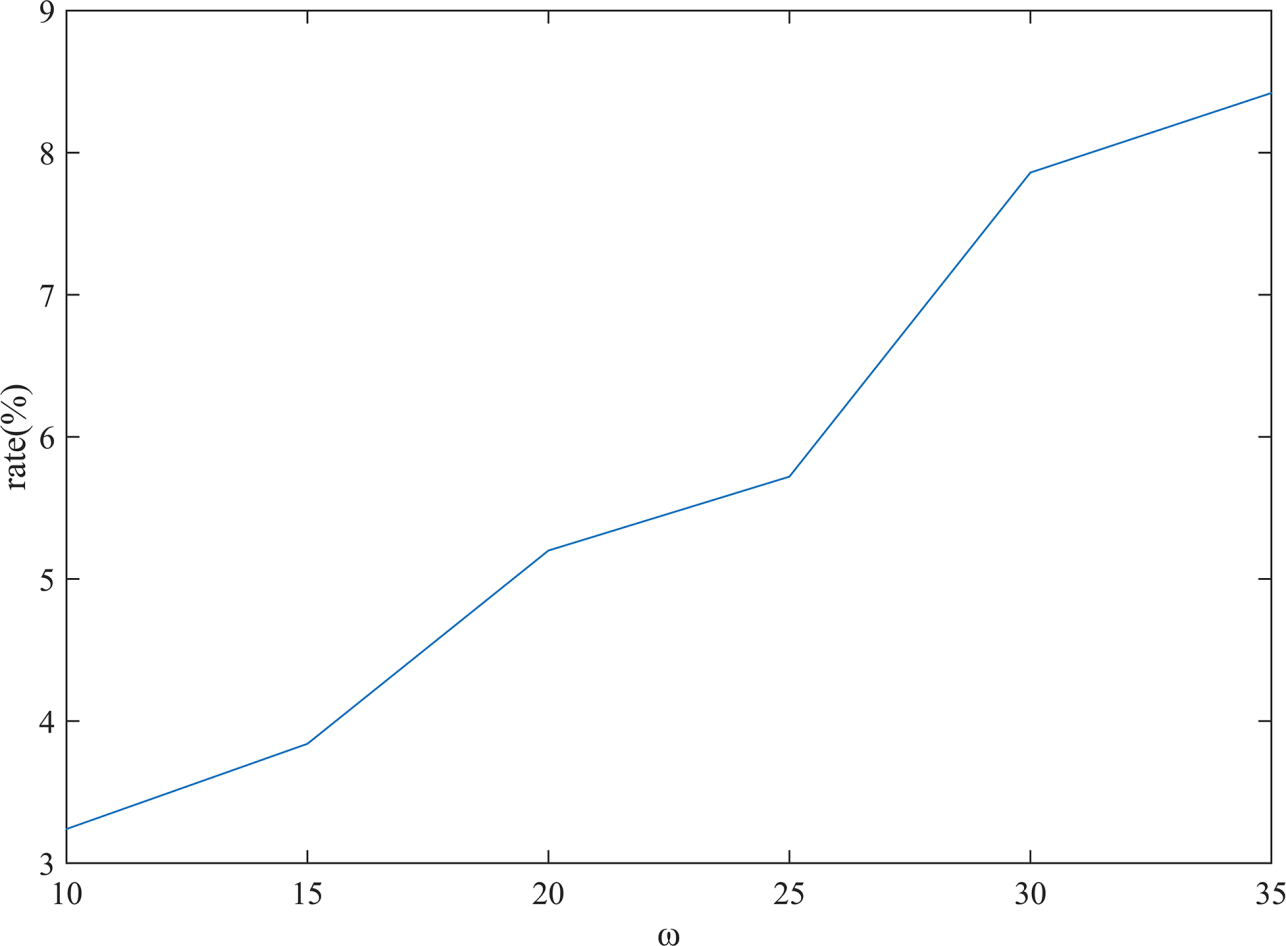
Proportion of road segments whose adjacent road segments are also in the same cluster.

The road segments that are adjacent to each other may have similar traffic characteristics; this property can be used instead of clustering for finding similar road segments. However, in contrast to our assumption, this is not very likely in practice. As shown in Fig 6, although the proportion of road segments whose adjacent segments are in the same cluster increases with ω, this proportion is still low. Therefore, it is more reasonable to find similar road segments by clustering rather than by adjacency relationships.

### Parameter estimation

**Fig 7:**
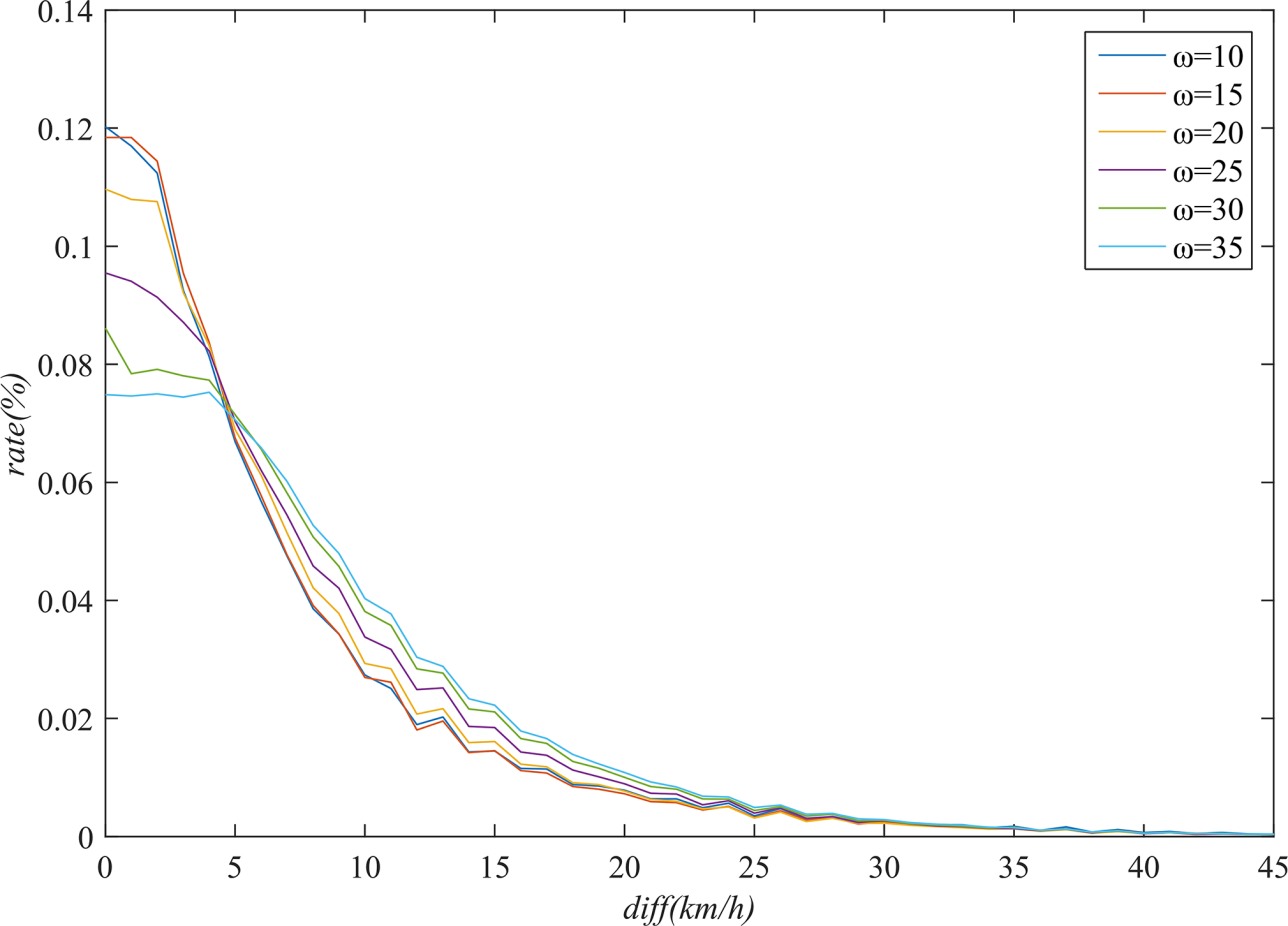
Distribution of diff in frequent cluster sets corresponding to different values of ω.

Statistical analysis of the distribution of *diff* was carried out in order to estimate the parameter *λ* in (13). In different frequent cluster sets corresponding to specific values of *ω*, the distribution of *diff* is different. In order to observe the distribution of *diff*, we calculated the ratio of each *diff* value to the total number of samples. As shown in Fig 7, the steepness of the distribution curve increases with *ω*, which indicates that the road segments in the frequent cluster generated on the basis of a smaller *ω* are more likely to have a higher degree of similarity, because the probability that *diff* takes a smaller value is higher.

The parameter *λ* was calculated as *1/E(diff)*, where *E(diff)* is the expectation of *diff*, and the equation was initialized with an initial parameter *λ* ^*^; then, the parameter was learned by iterative computation until it converged to a specific value. Table 3 lists *λ* values for six frequent cluster sets, *FCL = {FC*_*ω*_ *|ω = 10*, *15*, *20*, *25*, *30*, *35}*, as well as the sum of squared errors (SSE), root-mean-square error (RMSE), and R-square, which indicate that the equation works well for the samples.

**Table 3:** Estimated parameters of the emission probability equation corresponding to different frequent cluster sets.

*ω*	*λ*	SSE	R-square	RMSE
10	0.1337	0.000689	0.988	0.0030
15	0.1351	0.000847	0.986	0.0033
20	0.1251	0.000775	0.986	0.0032
25	0.1123	0.000694	0.986	0.0030
30	0.1012	0.000902	0. 980	0.0034
35	0.09267	0.001353	0. 966	0.0042

**Fig 8:**
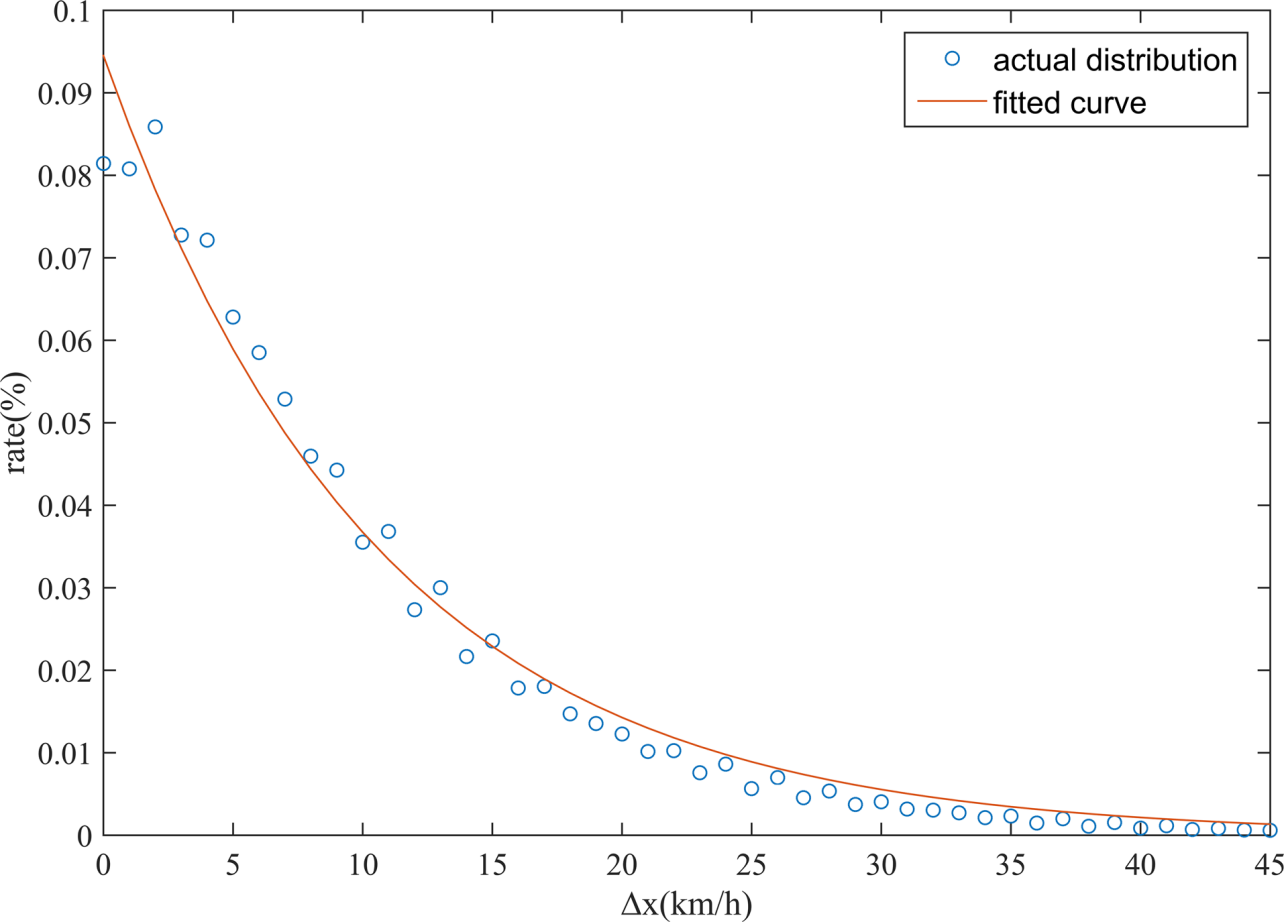
Distribution of the traffic state change *∆x*.

As shown in Fig 8, the traffic state change *∆x* follows an exponential distribution. The parameter *β* is calculated as *1/E(∆x)*, where *E(∆x)* is the expectation of *∆x*; after iterative computation, the estimated values of *β*, SSE, RMSE, and R-square are 0.09451, 0.000528, 0.002436, and 0.9871, respectively.

### Model accuracy and efficiency

A state sequence set of 8559 arterial road segments was prepared for testing. Because it is difficult to obtain the complete state set of these road segments, the states that were actually obtained were considered for accuracy analysis; the total number of these states, *N*_*base*_, was 6.19×10^5^. Among these states, a number of states were randomly selected as the missing states that need to be estimated. The missing state rate is denoted by *R*_*miss*_ *= N*_*miss*_ */N*_*base*_, where *N*_*miss*_ is the number of missing states. The mean absolute error (MAE) was employed to measure the estimation accuracy, and it is given by

$$MAE=\frac{1}{N_{estim}}\sum_{i=1}^{N_{estim}}\left|\hat{x_{i}}-x_{i}\right|$$ (16)

where *N*_*estim*_ is the number of states estimated, $\hat{x_{i}}$ is the estimated value of the *i*-th state, and *x*_*i*_ is the true value of the *i*-th state.

**Fig 9:**
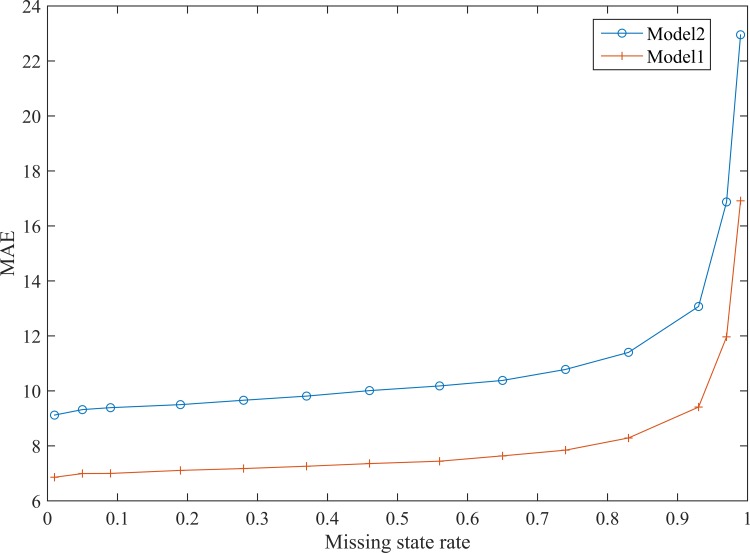
Comparison of accuracies of the two models.

The accuracies of two models, Model 1 and Model 2, were compared. Model 1 is the proposed model, which finds road segments with similar traffic states via clustering and frequent pattern mining, whereas Model 2 assumes that adjacent road segments have similar traffic conditions. As shown in Fig 9, MAE increases with *R*_*miss*_, and the MAE of Model 2 is significantly higher than that of Model 1, which implies that the proposed model is more accurate.

If there is no reference state, such as the previous state or the states of similar road segments, for the state *x* ^*r*,*t*^, the state cannot be estimated. The rate of the states that cannot be estimated is *R*_*miss*_ *-R*_*estim*_; then, the number of states that can be estimated is *R*_*valid*_ *= 1*-(*R*_*miss*_ *-R*_*estim*_), where *R*_*estim*_ = *N*_*estim*_ */N*_*base*_. As shown in Table 4, MAE increases gradually before *R*_*miss*_ reaches 83.33%, and *R*_*valid*_ remains high until *R*_*miss*_ reaches 92.59%, which indicates that the model is applicable to very sparse sample data.

**Table 4:** Estimation accuracy and coverage corresponding to different values of the missing state rate *R*_*miss*_.

*R_miss_*(%)	MAE(km/h)	*R_estim_*(%)	*R_valid_*(%)
0.93	6.86	0.92	99.99
4.63	6.99	4.61	99.98
9.26	7.01	9.2	99.94
18.52	7.11	18.24	99.72
27.78	7.18	26.77	98.99
37.04	7.26	35.88	98.84
46.3	7.36	44.93	98.63
55.56	7.44	53.92	98.36
64.81	7.64	62.8	97.99
74.07	7.84	71.51	97.44
83.33	8.29	79.41	96.08
92.59	9.41	85.12	92.53
97.22	11.96	82.05	84.83
99.07	16.91	64.58	65.51

**Fig 10:**
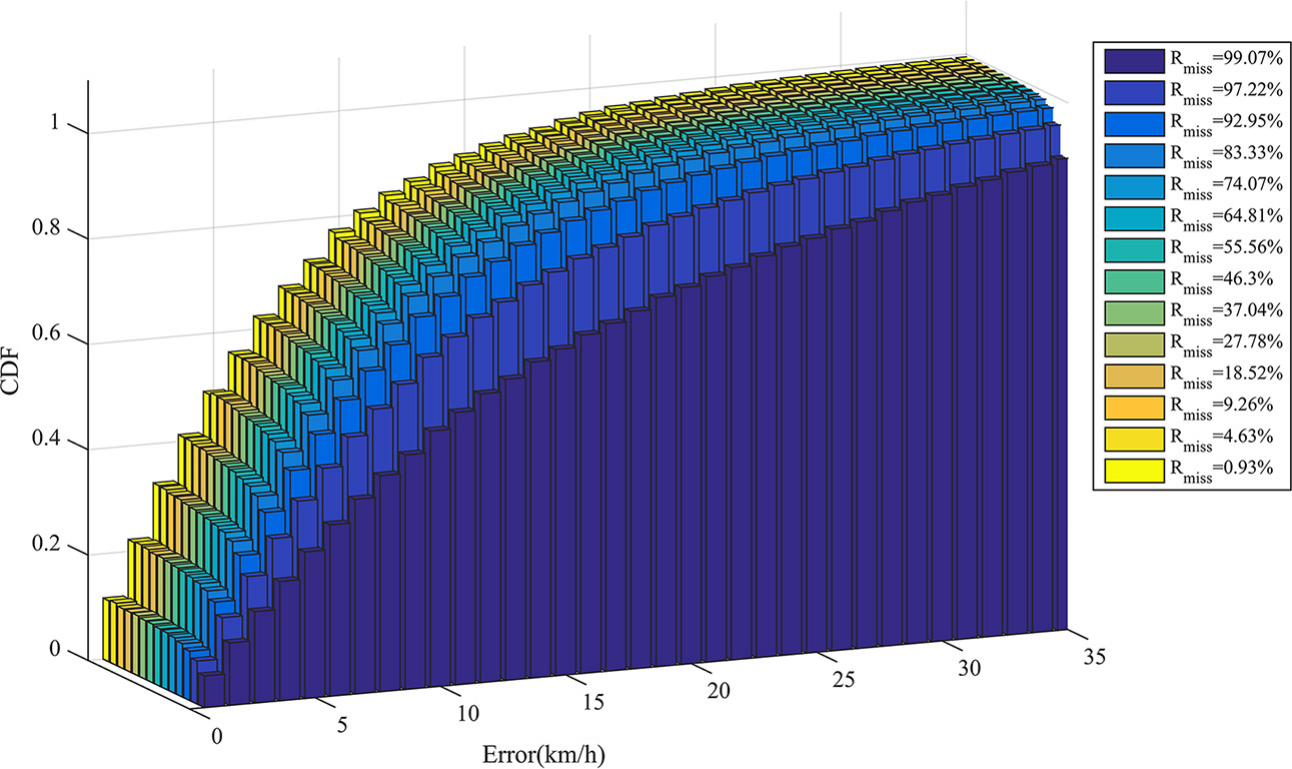
Estimation error CDF corresponding to different values of the missing state rate *R*_*miss*_.

The cumulative distribution function (CDF) of the estimation error *E* is given by

$$F_{E}(e)=P(E\leq e)=\frac{N(E\leq e)}{N_{estim}}$$ (17)

where estimation error *E* is the absolute value of the difference between the estimated and observed values, *N*_*estim*_ is the number of estimated states, and *N(E ≤ e)* is the number of estimated states whose error is less than or equal to *e*. Fig 10 shows the CDFs of estimation errors corresponding to different values of the missing state rate *R*_*miss*_. Before *R*_*miss*_ reaches 83.33%, the CDF curve is steeper, which indicates that most errors are small. For example, when *R*_*miss*_ = 83.33%, more than 52.79% of the errors are less than or equal to 5 and more than 76.88% of the errors are less than or equal to 10.

The states that are obtained are considered as the true states; then, the estimated error distribution function in the global scope is given by

$$F^{\prime }(e)={1-R_{miss}+F}_{E}(e)∙R_{estim}$$ (18)

Table 5 summarizes the global distribution of the estimated error, which reflects the accuracy of the model corresponding to different values of the missing state rate *R*_*miss*_. According to the error distribution, it is easy to determine whether the accuracy of the model meets the requirements of the application. For example, in an application that requires 90% of the errors are to be less than 5 km/h, if the missing state rate is less than 18.52%, then the model may be suitable for the application.

**Table 5:** Global cumulative distribution of the estimated error corresponding to different values of the missing state rate *R*_*miss*_.

*R_miss_*(%)	*F'(5)*(%)	*F'(10)* (%)	*F'(20)* (%)	*F'(30)* (%)
0.93	99.59	99.81	99.96	99.98
4.63	97.96	99.06	99.79	99.94
9.26	95.90	98.09	99.55	99.86
18.52	91.66	95.98	98.92	99.54
27.78	87.06	93.43	97.75	98.70
37.04	82.66	91.26	97.16	98.47
46.30	78.22	89.00	96.45	98.12
55.56	73.71	86.61	95.66	97.68
64.81	68.84	83.9	94.64	97.10
74.07	63.68	80.91	93.32	96.28
83.33	57.43	76.58	90.79	94.41
92.59	48.53	68.92	84.88	89.67
97.22	37.74	56.32	72.91	79.33
99.07	21.94	34.32	48.19	56.34

**Fig 11:**
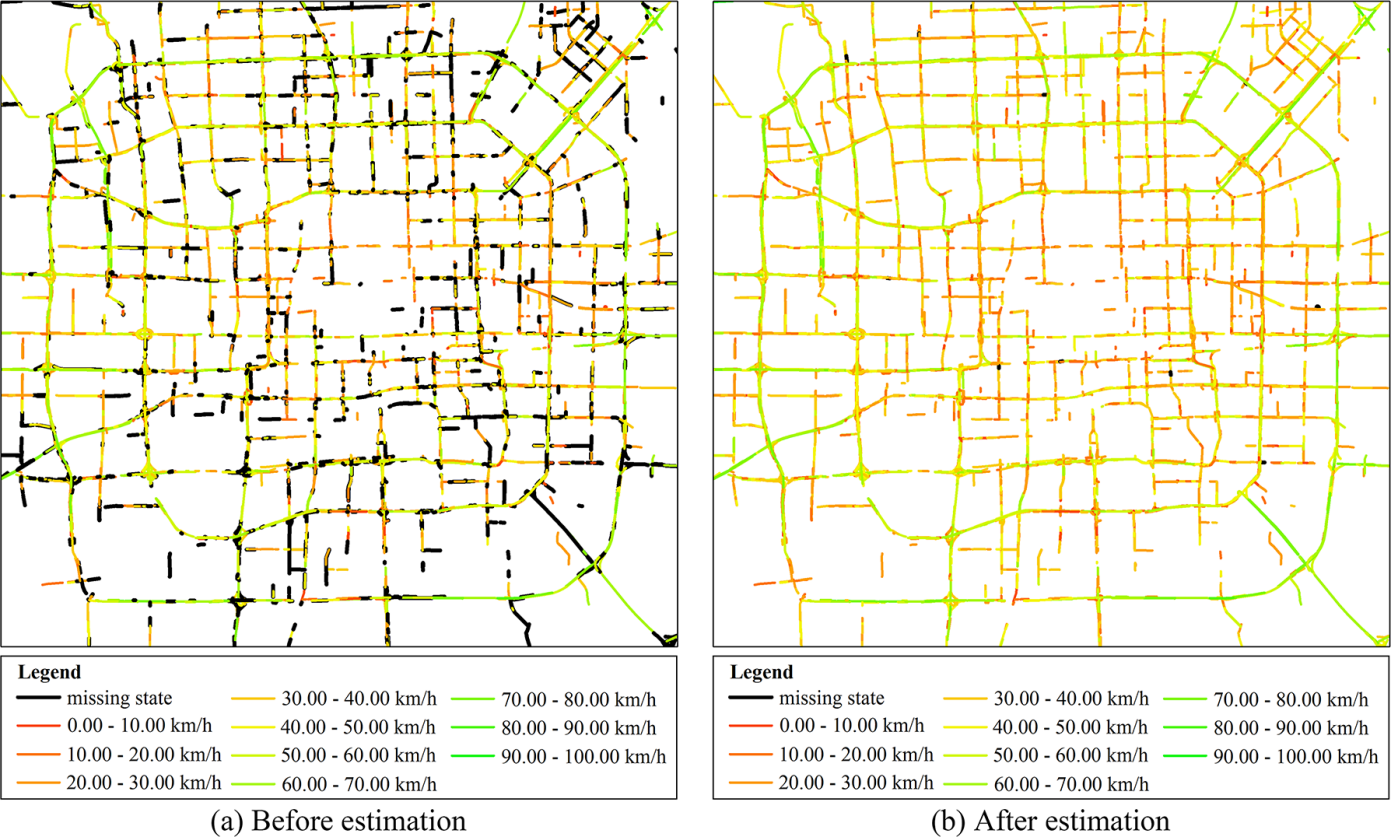
Traffic condition of arterials in Beijing at 14:20–14:30. (a) Traffic condition map before estimation, and (b) traffic condition map after estimation; the black regions represent missing states.

In our data source, the missing state rate of the arterial roads was around 33%, while the missing state rate of the other roads was around 65%. Samples of 8559 arterial roads were considered. The error was less than or equal to 5 (resp. 10) for more than 84.84% (resp. 92.61%) of the states; this indicates a high estimation accuracy. Fig 11(A) shows the traffic state map of the arterial roads in Beijing at the 50^th^ time slot (14:10–14:20) before estimation, and Fig 11(B) shows the states of the roads after estimation. Most of the missing states were estimated, and the estimated values were very close to the true values.

The main factor that affects the efficiency of the model is the number of candidates for the hidden states, *N*_*cand*_, which has been discussed in Section 3.4. Several values of *N*_*cand*_ were selected for the experiments, where the missing state rate was around 74%. The results show that the accuracy improved as *N*_*cand*_ increased; however, the time cost increased significantly (Table 6). When *N*_*cand*_ reached around 12, the accuracy stabilized and the model could estimate approximately 4169.22 states per second. From the viewpoint of practical application, the model can meet the efficiency requirements of metropolitan real-time traffic estimation.

**Table 6:** Efficiency of the model corresponding to different values of *N*_*cand*_.

*N_cand_*	MAE (km/h)	cost (s)	speed (states/s)
4	8.35	21	19456.38
6	7.9	25	16343.36
8	7.82	52	7857.38
10	7.78	66	6190.66
12	7.76	98	4169.22
14	7.76	130	3142.95
16	7.76	154	2653.14
18	7.76	213	1918.23
20	7.76	243	1681.41

## Conclusion

This paper presented an effective and efficient HMM-based model for urban-scale traffic estimation using floating car data. Clustering analysis and pattern mining were adopted to analyze a large data set of real probe data collected from a fleet of 12,600 taxis in Beijing, China, and it was found that there exist frequent clusters in which the road segments have similar traffic characteristics. Comparative analysis showed that the model based on clustering is more effective than the model based on adjacency relationships for traffic estimation. In order to achieve a trade-off between clustering accuracy and coverage, a multi-clustering strategy was adopted in the estimation process. Experimental results showed that the model can be applied to different scenarios; even when more than 70% of the original data are missing, the model can guarantee that more than 80% of the states have relatively small errors. In addition, the model was implemented using a real-time algorithm, which offers higher precision and has a broader scope for application than some offline traffic estimation algorithms.
